# Case Report: Cytomegalovirus-associated granulomatous lung disease in an immunocompetent preterm infant

**DOI:** 10.3389/fimmu.2026.2000675

**Published:** 2026-10-09

**Authors:** Yiping Zhou, Yaru Zhang, Rufang Zhang, Yucai Zhang, Yun Cui

**Affiliations:** 1 Department of Pediatric Intensive Care Unit, Shanghai Children’s Hospital, Shanghai Jiao Tong University School of Medicine, Shanghai, China; 2 Department of Cardiothoracic Surgery, Shanghai Children’s Hospital, Shanghai Jiao Tong University School of Medicine, Shanghai, China

**Keywords:** cytomegalovirus, granulomatous lung disease, metagenomic next-generation sequencing, postnatally acquired infection, preterm infant, video-assisted thoracoscopic surgery

## Abstract

Granulomatous lung disease caused by cytomegalovirus (CMV) has not been described in immunocompetent infants. We report a 5-month-old boy, born at 31^+5^ weeks of gestation, with a 2-month history of isolated cough and normal growth. Contrast-enhanced chest computed tomography showed a multiloculated cystic mass in the right upper lobe. CMV DNA was detected in whole blood (1.41 × 10^4^ copies/mL) and urine (1.44 × 10^6^ copies/mL). Intravenous ganciclovir for 2 weeks produced no radiological change; because a tumor could not be excluded, video-assisted thoracoscopic right upper lobectomy was performed. Histology showed extensive necrosis with well-formed epithelioid granulomas and multinucleated giant cells. Stains for acid-fast bacilli and fungi and Epstein-Barr virus-encoded RNA *in situ* hybridization were negative. To exclude an infectious granulomatous disease, metagenomic next-generation sequencing (mNGS) of the resected tissue identified CMV at 1,143 reads per million as the only pathogen. The postoperative course was uncomplicated, with no recurrence at 6 months. CMV produced a localized granulomatous mass mimicking a congenital lung lesion or neoplasm, and tissue mNGS provided the decisive microbiological evidence. When a preterm infant has an unexplained chronic pulmonary lesion, CMV should be considered, and mNGS of pathological tissue can establish the diagnosis.

## 1 Introduction

CMV is one of the most common congenital and perinatal viral infections, and seroprevalence varies widely between regions, from about 40% to 100% (1, 2). Most immunocompetent children who become infected have no symptoms or a self-limiting illness that resembles infectious mononucleosis (1). The situation differs in preterm infants, immunocompromised patients and congenitally infected newborns, in whom CMV may involve the lungs, liver, central nervous system and hematopoietic system (3).

Granulomatous lung disease is a heterogeneous group of conditions defined by granuloma formation in the pulmonary parenchyma. Sarcoidosis, hypersensitivity pneumonitis, mycobacterial and fungal infection, and drug reactions are the recognized causes (4). CMV has occasionally been associated with granulomatous inflammation in immunocompromised adults, mostly in patients with acquired immunodeficiency syndrome or after transplantation (5). We are not aware of a previous report of CMV-associated granulomatous lung disease in an immunocompetent infant.

We describe a preterm infant who presented with a prolonged cough and an isolated cystic mass in the right upper lobe. The diagnosis was established by mNGS of the resected lesion, an approach that has proved useful for pathogen detection in pathological tissue (6).

## 2 Case description

A 5-month-old boy was admitted to our pediatric ward for evaluation of a persistent lesion in the right upper lobe. He was born at 31^+5^ weeks of gestation (G2P1; the first pregnancy ended in spontaneous abortion) with a birth weight of 2,150 g. Respiratory distress syndrome, neonatal pneumonia, hyperbilirubinemia and a patent foramen ovale complicated the neonatal period; he required mechanical ventilation in the neonatal intensive care unit (NICU) and was discharged after improvement. Quantitative real-time PCR for CMV DNA in blood and urine during the neonatal admission was negative, and the chest radiograph obtained at that time showed no lesion in either lung, which argued against a congenital infection and pointed to presumed postnatally acquired CMV (pCMV).

For the next 2 months he coughed almost every day. He had no fever and no tachypnea, and he fed, slept and gained weight normally. A chest radiograph taken at 5 months of age, before CT, showed consolidation in the right upper lobe, and chest computed tomography (CT) at another hospital then showed a lesion in the same lobe. He received intravenous ganciclovir for 2 weeks. The lesion did not change on repeat radiography or CT, and he was referred to our hospital.

On admission, his temperature was 36.8 °C, pulse 138 beats/min, respiratory rate 26 breaths/min, blood pressure 98/65 mmHg and oxygen saturation 98% while breathing room air. He weighed 7.5 kg and was 64 cm long. Breath sounds were coarse over the right upper lung field, without crackles or wheezes. The liver, spleen and lymph nodes were not enlarged, and the skin was normal. **Table 1** summarizes the course of the illness.

**Table 1:** Timeline of the episode of care.

Time point	Clinical events and findings	Interventions and outcomes
Birth (31 ^+5^ weeks; 2,150 g)	Respiratory distress syndrome, neonatal pneumonia, hyperbilirubinemia, patent foramen ovale	Mechanical ventilation in the NICU; discharged after improvement
Neonatal admission	Neonatal CMV DNA PCR negative; chest radiograph without a space-occupying lesion	Congenital infection considered unlikely; pCMV suspected
About 3 months of age	Daily cough for 2 months, no fever or tachypnea; chest CT showing a right upper lobe lesion	Intravenous ganciclovir for 2 weeks; no change on imaging
5 months of age (admission)	Normal vital signs; WBC 20.27 × 10 ^9^/L, CRP 5 mg/L; CMV DNA in whole blood 1.41 × 10 ^4^, plasma 1.17 × 10 ^4^, urine 1.44 × 10^6^ copies/mL; chest radiograph showing consolidation in the right upper lobe; repeat CT showing a multiloculated cystic lesion	BALF targeted sequencing: CMV 57 × 10^3^ copies/mL; tumor not excluded, referred for surgery
5 months of age (surgery)	Dense adhesions and necrotic material at operation; histology showing necrotizing granulomas; tissue mNGS showing CMV 1,143 RPM as the only pathogen	VATS right upper lobectomy, diagnostic and therapeutic
Postoperative period	Unremarkable recovery	Discharged on postoperative day 5
6-month follow-up	No symptoms, normal growth, no recurrence on imaging	No further treatment

### 2.1 Diagnostic assessment

The white blood cell count was 20.27 × 10^9^/L (neutrophils 28.1%, lymphocytes 61.1%), hemoglobin 116 g/L, platelet count 440 × 10^9^/L and C-reactive protein 5 mg/L. Lymphocyte subsets were elevated: CD3^+^ 67.6%, CD4^+^ 46.6%, CD8^+^ 25.1% and CD19^+^ 17.7%. Serum interleukin-6 (IL-6) and tumor necrosis factor-α (TNF-α) were mildly raised (IL-6: 7.93 pg/mL; TNF-α: 11.53 pg/mL). Liver and renal function tests were normal. Cranial magnetic resonance imaging showed slight widening of the bilateral temporal extra-axial spaces and was otherwise normal; hearing assessment showed normal auditory brainstem responses, and ophthalmological examination showed no abnormalities. He had no history of recurrent or severe infection and no other features to suggest an underlying immunodeficiency.

Quantitative PCR for CMV DNA was positive in whole blood (1.41 × 10^4^ copies/mL), plasma (1.17 × 10^4^ copies/mL) and urine (1.44 × 10^6^ copies/mL), TORCH screening was performed, and CMV IgG and IgM were positive. At bronchoscopy, targeted next-generation sequencing of bronchoalveolar lavage fluid (BALF) identified CMV at 57 × 10^3^ copies/mL. Repeat contrast-enhanced CT showed a multiloculated cystic lesion occupying the right upper lobe (**Figure 1**). Congenital pulmonary airway malformation (CPAM) with secondary infection and a neoplastic lesion were both considered possible.

**Figure 1:**
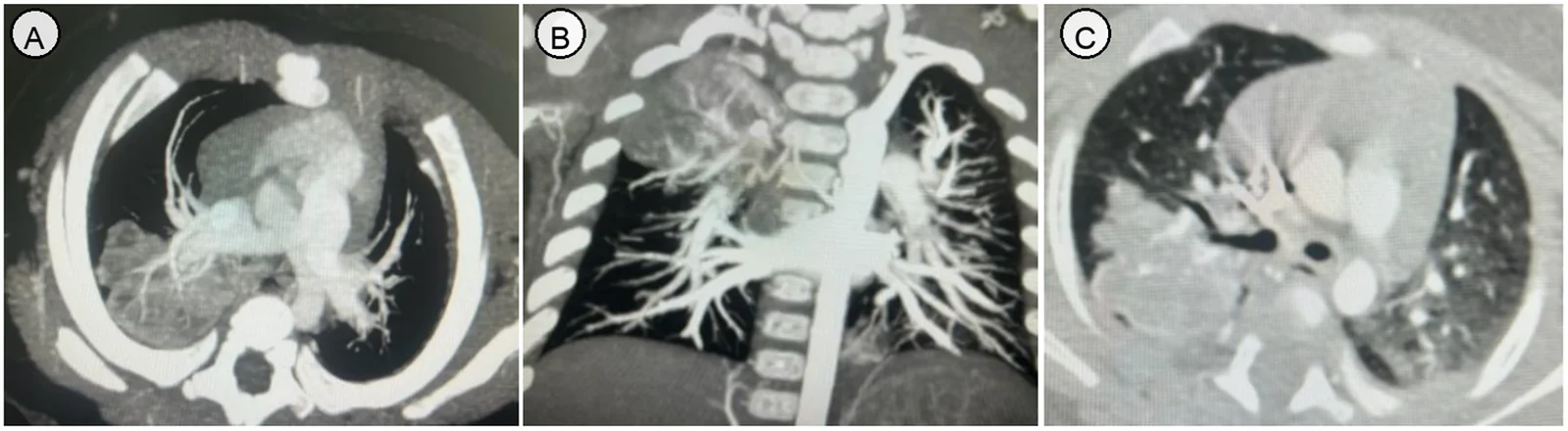
Contrast-enhanced chest CT showing a multiloculated cystic mass in the right upper lobe. **(A, B)** Display sequence; **(C)** lung window. CPAM with secondary infection was considered most likely, although a neoplastic lesion could not be excluded.

Because the lesion had not responded to antiviral treatment and a tumor could not be excluded, VATS right upper lobectomy was performed. Dense adhesions and necrotic material were found at operation. Histology showed extensive necrosis with well-formed granulomas composed of epithelioid histiocytes and multinucleated giant cells, together with acute and chronic inflammatory infiltrates (**Figure 2**). Immunohistochemistry was positive for CD163, CD68 and LCA, with focal high Ki-67 expression, whereas CK, SMA and TTF-1 were negative in the lesional areas. Stains for AFB and fungi (PAS, PASM and fungal fluorescence) were negative, as was *in situ* hybridization for EBER. An interferon-γ release assay (T-SPOT.TB) on peripheral blood was negative. Tuberculosis was therefore considered excluded on the basis of the negative acid-fast stain of the lesion, the negative T-SPOT.TB result and the absence of mycobacterial sequences in the tissue mNGS. mNGS of the resected tissue, performed to identify an infectious cause of the granulomatous inflammation, detected CMV at 1,143 reads per million (RPM) and no other pathogen.

**Figure 2:**
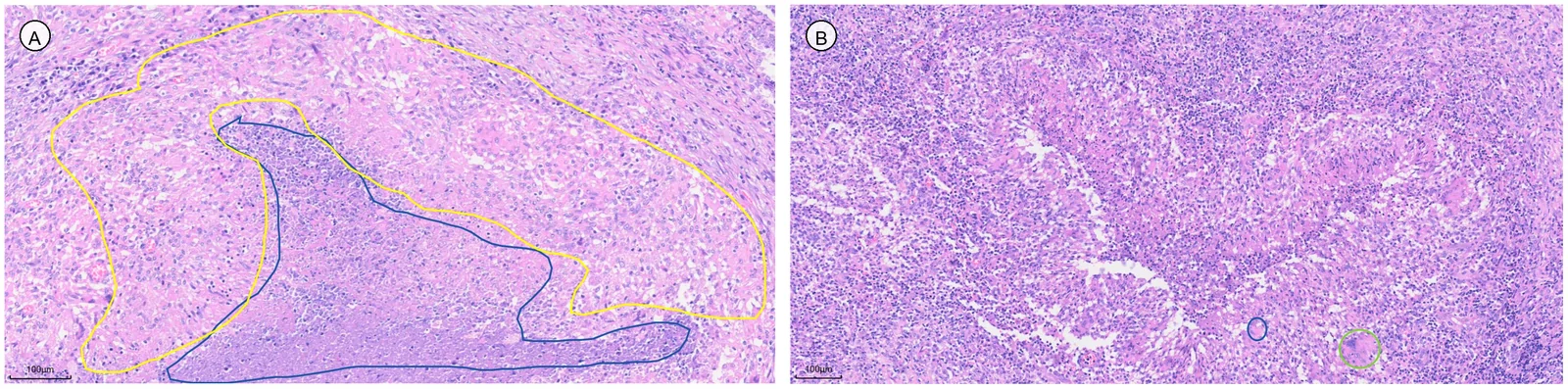
Histopathology of the resected right upper lobe. **(A)** Hematoxylin and eosin (H&E) staining, ×200. The area outlined in blue is necrotic tissue; the area outlined in yellow shows the granulomatous architecture, with aggregates of proliferating histiocytes, including epithelioid cells and multinucleated giant cells, forming well-circumscribed nodules. **(B)** H&E staining, ×150. Blue circle, an epithelioid cell; green circle, a multinucleated giant cell.

### 2.2 Therapeutic intervention

Treatment was given in three steps. Intravenous ganciclovir was given for 2 weeks at the referring hospital and then stopped, because the lesion had not changed on imaging. This infant had no respiratory distress, no hypoxemia, no hepatitis, no cytopenia and no evidence of CMV disease in other organs; as the infection caused no symptoms and no end-organ damage, antiviral treatment was not required. Current recommendations advise antiviral therapy for infants with symptomatic CMV disease or end-organ involvement and do not support treatment of asymptomatic infection (7, 8). Urine CMV DNA was not re-tested during the clinical course, because the infant remained asymptomatic and treatment decisions were based on the clinical and imaging findings rather than on viral load. VATS right upper lobectomy followed, which served both to establish the diagnosis and to remove the necrotizing granulomatous mass. After surgery he received nebulized budesonide and no antibiotics, as there was no evidence of bacterial infection. Recovery was uneventful.

### 2.3 Follow-up and outcomes

The boy was discharged on the fifth postoperative day. At follow-up 6 months later he was free of symptoms, growing normally, and imaging showed no recurrence. Regular outpatient follow-up with clinical assessment and periodic imaging is planned to detect any late recurrence.

## 3 Discussion

To our knowledge, this is the first report of CMV-associated granulomatous lung disease in an immunocompetent infant. We searched PubMed/MEDLINE on 23 September 2026 without date or language restriction, combining terms for the virus (cytomegalovirus[tiab] OR CMV[tiab] OR “Cytomegalovirus”[MeSH]) with terms for granulomatous inflammation (granuloma[tiab] OR granulomatous[tiab] OR “Granuloma”[MeSH]) and terms for the lung (lung[tiab] OR pulmonary[tiab] OR “Lung”[MeSH]); 33 records were retrieved. Restricting the same set to infants and neonates (infant[tiab] OR neonate[tiab] OR newborn[tiab] OR preterm[tiab] OR “Infant”[MeSH]) left a single record, a neuropathological series of patients with acquired immunodeficiency syndrome, and no report of CMV-associated granulomatous lung disease in an immunocompetent infant was identified. The presentation was indolent. Over 2 months the only symptom was cough, and the infant continued to thrive. Imaging showed a localized cystic mass rather than the diffuse ground-glass opacities or small nodules usually seen in CMV pneumonia in neonates and infants (3). For this reason CPAM with infection, pulmonary sequestration and tumors such as inflammatory myofibroblastic tumor were considered first. However, the resected lesion showed necrotizing granulomatous inflammation rather than the cystic architecture typical of CPAM, and tissue mNGS identified no pathogen other than CMV, both of which argue against infected CPAM as the cause of the lesion. Moreover, no lesion was visible in the right upper lung on the chest radiograph taken during the neonatal admission, whereas a congenital malformation such as CPAM should have been present from birth; for these reasons CPAM was not considered further. A solitary pulmonary nodule caused by CMV has been described once before, in an adult receiving chemotherapy for lymphoma (5). Our experience suggests that CMV should be added to the list of possible causes of a solitary lung lesion in an infant, particularly one born preterm.

Mycobacteria, fungi and parasites are the usual infectious causes of chronic granulomatous change in the lung (4), and CMV is an uncommon one, especially in an immunocompetent host. Focal tumor-like lesions attributed to CMV have also been reported in the liver of an immunocompetent adult (9). Several findings in this case support a causal role for CMV. mNGS was performed on the resected lesion itself and detected CMV at 1,143 RPM as the only pathogen, which argues against contamination or co-infection. Other granuloma-forming causes were excluded by negative AFB and fungal stains, negative EBER *in situ* hybridization and negative markers of malignancy, leaving a necrotizing granulomatous pattern consistent with a local immune response to persistent viral antigen. Sarcoidosis was also considered unlikely: it is exceedingly rare in infancy, its granulomas are typically non-necrotizing, whereas the granulomas in this case were necrotizing, and there was no evidence of systemic involvement. Active CMV infection was also confirmed independently by viral loads in blood, urine and BALF. Despite viremia and viruria, there were no signs of systemic disease and no involvement of other organs at any time; the infection remained localized to the pulmonary lesion, an indolent course consistent with containment of the virus by the host response. CMV persists despite a vigorous cell-mediated response: it interferes with cGAS-STING signaling and type I interferon production through UL31, UL42 and UL83, reduces expression of ligands for NK cell-activating receptors through UL16 and UL141, and impairs MHC class I presentation through US2 and US11 (10). These considerations are hypothetical: we propose, as a hypothesis for further study rather than a conclusion from this case, that granuloma formation represents one way in which the host contains a virus that it cannot clear, as it does in tuberculosis and fungal infection. The elevated lymphocyte subsets and mildly raised IL-6 and TNF-α in this infant would be compatible with such an activated immune response.

Several limitations should be noted. This is a single case. CMV was not measured in maternal serum or breast milk, and the route of transmission therefore remains unknown, although breast milk is the main source of pCMV in preterm infants (11). Urine CMV DNA was not re-tested after treatment, so the clearance of urinary viral shedding was not documented. The immunological work-up included lymphocyte subsets, cytokines, humoral immunity (immunoglobulins), complement and neutrophil function, and the results were normal. Apart from the present episode the infant had no clinical or laboratory evidence of immunodeficiency, so we use the term immunocompetent in its clinical sense.

In full-term infants pCMV is usually mild or silent. In preterm infants, and especially those with very low birth weight, it can cause a sepsis-like illness and lead to pulmonary and neurodevelopmental injury, hepatic dysfunction, bone marrow suppression, hearing loss and retinopathy (12). Among CMV-seropositive mothers who breastfeed preterm infants, reactivation during lactation occurs in up to 96%, about 37% of infants acquire CMV from milk, and 12% develop sepsis-like symptoms (11). Our patient received mixed feeding. When a preterm infant has an unexplained chronic pulmonary lesion or unexplained systemic inflammation, pCMV should be considered, and measures such as CMV screening of breast milk, pasteurization or the use of previously frozen milk can reduce transmission (12).

In this infant CMV produced an isolated granulomatous lesion that mimicked a tumor; resection was diagnostic and was followed by clinical and radiological resolution, with an excellent outcome at 6 months of follow-up. Reports such as this one help to define the range of presentations of CMV in immunocompetent children.

## 4 Patient perspective

The parents said that the persistent cough and the uncertainty about the lung lesion had been difficult for the family. After the operation and the confirmation of a treatable, non-malignant cause, they were relieved, and at follow-up they described the boy’s growth and development as normal.

## Data Availability

The datasets presented in this article are not readily available because of ethical and privacy restrictions. Requests to access the datasets should be directed to the corresponding author.
